# Evidence From Ghana Indicates That Childhood Cancer Treatment in Sub-Saharan Africa Is Very Cost Effective: A Report From the Childhood Cancer 2030 Network

**DOI:** 10.1200/JGO.17.00243

**Published:** 2018-05-03

**Authors:** Lorna Renner, Shivani Shah, Nickhill Bhakta, Avram Denburg, Sue Horton, Sumit Gupta

**Affiliations:** **Lorna Renner**, University of Ghana School of Medicine and Dentistry, Accra, Ghana; **Shivani Shah**, A**vram Denburg**, **Sue Horton**, and **Sumit Gupta**, Hospital for Sick Children, Toronto; **Sue Horton**, University of Waterloo, Waterloo, Ontario, Canada; and **Nickhill Bhakta**, St Jude Children’s Research Hospital, Memphis, TN.

## Abstract

**Purpose:**

No published study to date has examined total cost and cost-effectiveness of maintaining a pediatric oncology treatment center in an African setting, thus limiting childhood cancer advocacy and policy efforts.

**Methods:**

Within the Korle Bu Teaching Hospital in Accra, Ghana, costing data were gathered for all inputs related to operating a pediatric cancer unit. Cost and volume data for relevant clinical services (eg, laboratory, pathology, medications) were obtained retrospectively or prospectively. Salaries were determined and multiplied by proportion of time dedicated toward pediatric patients with cancer. Costs associated with inpatient bed use, outpatient clinic use, administrative fees, and overhead were estimated. Costs were summed for a total annual operating cost. Cost-effectiveness was calculated based on annual patients with newly diagnosed disease, survival rates, and life expectancy.

**Results:**

The Korle Bu Teaching Hospital pediatric cancer unit treats on average 170 new diagnoses annually. Total operating cost was $1.7 million/y. Personnel salaries and operating room costs were the most expensive inputs, contributing 45% and 21% of total costs. Together, medications, imaging, radiation, and pathology services accounted for 7%. The cost per disability-adjusted life-year averted was $1,034, less than the Ghanaian per capita income, and thus considered very cost effective as per WHO-CHOICE methodology.

**Conclusion:**

To our knowledge, this study is the first to examine institution-level costs and cost-effectiveness of a childhood cancer program in an African setting, demonstrating that operating such a program in this setting is very cost effective. These results will inform national childhood cancer strategies in Africa and other low- and middle-income country settings.

## BACKGROUND

Among children diagnosed with cancer in high-income countries (HICs), long-term cure rates are now > 80%.^1^ Nearly 90% of the global pediatric population resides in low- and middle-income countries (LMICs), where childhood cancer survival rates vary between 10% and 50%.^2,3^ Twinning programs involving financial and infrastructural support from HIC institutions have resulted in significant survival improvements in individual LMIC centers.^4^ Improving population-based LMIC childhood cancer outcomes will instead require regional and national childhood cancer strategies that conform to local health system contexts and public resources available.^5,6^

A major barrier to creating and implementing national childhood cancer strategies is a paucity of data on the cost of delivering childhood cancer treatment. It is commonly believed that LMIC health systems are unable to bear the costs of pediatric oncology services. Although recent data suggest this assumption is inaccurate,^7,8^ there is scant evidence on the financial and economic costs of treating childhood cancer in LMICs. Without cost data, policymakers have little context-relevant evidence to inform the creation or expansion of childhood cancer services. Indeed, assumptions that childhood cancer treatment is expensive may prevent policymakers from even considering pediatric oncology when setting national health priorities. Studies of the cost and cost-effectiveness of such treatment in LMIC settings are therefore essential.

A preliminary analysis of theoretical cost-effectiveness thresholds suggested that childhood cancer treatment may be financially feasible in LMICs but was limited to two specific malignancies and neglected nondrug costs.^7,9^ A recent center-level study by our team in El Salvador represented the first rigorous and comprehensive LMIC childhood cancer costing analysis, to our knowledge, finding that a childhood cancer treatment unit represented a very cost-effective intervention.^8^ Whether analogous treatment units in sub-Saharan Africa, with more limited resources and generally lower survival rates, are also cost effective is unknown.

Our objective was to determine the total cost of maintaining a major pediatric cancer treatment unit in a sub-Saharan setting. Using administrative and clinical data from the Korle Bu Teaching Hospital (KBTH) in Accra, Ghana, we also aimed to determine the cost-effectiveness of the program.

## METHODS

### Study Setting

KBTH is the largest hospital in Ghana (2,000 beds) and the third largest in sub-Saharan Africa, serving a catchment area of approximately 19.74 million in the southern half of the country. In addition to Komfo Anoye in Kumasi, KBTH is one of two Ghanaian hospitals with the facilities to treat childhood cancer, diagnoses approximately 170 new cases of cancer in patients younger than 14 years of age annually, and contains 30 inpatient beds. An outpatient clinic sees an average of 77 patients per day. Forty-one full-time-equivalent medical personnel were involved in the care of pediatric oncology patients, including two pediatric oncologists and 21 nurses. Patients deemed at high risk of complications are kept as inpatients for close monitoring, including patients presenting with bulky disease. Because of limited pediatric oncology services in the West African region, KBTH also admits patients from neighboring countries. In 2016, approximately 45% of patients were diagnosed with leukemia or lymphoma, 42% with solid tumors, and 13% with CNS tumors.^10,11^

Treatment protocols are based on international standards but are often modified to account for greater risk of toxicity or lack of resources. For example, for children with acute lymphoblastic leukemia, a modified UKALL protocol is used; induction doses of anthracycline are often omitted to prevent toxicity.

The pediatric oncology program is primarily financed by the Ministry of Health. Although a National Health Insurance Authority exists in Ghana, it does not cover all medications and services, meaning that families must absorb these costs. For example, although common generic antibiotics are covered, chemotherapy for childhood cancer is not, nor are diagnostic tests such as computed tomography scans or pathology. Private philanthropic sources of funding exist to offset out-of-pocket costs incurred by families; the most prominent are World Child Cancer, an international nongovernmental agency, and the Ghana Parents’ Association for Childhood Cancer. Local private and faith-based organizations also play an important role in the day-to-day operation and financing of the pediatric oncology program, including fundraising and providing financial assistance to low-income families for transportation, meals, and medical services.

### Data Collection

To collect cost data, a detailed abstraction tool was developed after compartmentalizing costs into: personnel (both medical and support), room and board for patients and their families (hoteling), outpatient clinic, shared services (pharmacy, pathology, surgery, radiation, imaging, and blood bank), other services (information technology, training), and other central hospital services (utilities, human resources, administrative costs). The structure of the abstraction tool is available in Appendix Table A1. All costs were collected and included regardless of funding stream (ie, government *v* family out of pocket *v* philanthropic).

Information on the volume and unit cost of items came from various sources. Medical personnel costs were determined by multiplying salary figures for relevant health care providers by the self-reported proportion of their time dedicated to pediatric oncology care. Operating room (OR) costs associated with pediatric oncology patients were determined by obtaining OR records for a 4-week period within the last calendar year and determining the number of OR hours used by pediatric oncology patients. OR hours were categorized as major versus minor on the basis of the length of time in surgery (ie, > 1 hour *v* ≤ 1 hour) and by the type of surgery (procedures involving extensive resections, thoracotomies, CNS, or cardiopulmonary procedures were all considered major). Total hours were multiplied by 13 to derive an annual figure and then multiplied by the cost of an average hour of OR time in Ghana as determined by the WHO (stratified by major *v* minor) to provide an estimate of the annual OR budget attributable to children with cancer.^12^

The pediatric cancer unit at KBTH does not maintain financial records separate from those of the overall hospital. Thus, for a number of items, including diagnostic imaging, radiation, and blood products, four 1-week periods within the prior calendar year were randomly chosen and patient charts reviewed to record all of the above services delivered to pediatric oncology patients. Unit costs were obtained from the appropriate hospital department. Unit costs and average number of items ordered over each 1-week period were also multiplied by 13 to derive estimated annual utilization figures.

The number and types of laboratory tests and medications (supportive and chemotherapeutic) ordered for pediatric oncology patients were recorded prospectively for 2 weeks; both inpatients and outpatients were included. Unit costs were obtained from appropriate hospital departments and, in the case of medications, adjusted based on dosage. Unit costs for diagnostic services incorporated the costs of personnel (eg, laboratory technicians) inherent in providing the service. Unit costs and volumes were multiplied to determine the total laboratory and medication-associated cost of treating children with cancer over the 2 weeks and then multiplied by 26 to determine the annual costs.

Information on the time devoted by nonmedical personnel (eg, clerical staff) to pediatric oncology services was unavailable. Such services included registration of patients in the inpatient and outpatient clinics, data entry into the cancer registry, and other clerical, technical, and administrative tasks. The unit is also supported by the central administration for activities such as human resources, legal activities, communications, and relationships with external organizations and government. In the absence of nonmedical personnel cost, we thus used the same ratio of cost of nonmedical to medical personnel (25:75) as for the Pediatric Cancer Department at the Hospital Nacional de Niños Benjamin Blum, El Salvador, which maintains separate financial statistics for their pediatric cancer unit and thus produced, to our knowledge, the first published estimates of the cost of running a pediatric cancer unit in an LMIC.^8^ For the cost of central administration, we again used data from Hospital Nacional de Niños Benjamin Blum, which, by prorating the cost of utilities and central administration by the pediatric cancer unit’s share of inpatient admissions, was able to determine that such costs came to 11.8% of total cost of the pediatric cancer unit. Such assumptions were necessary to include some estimate of administrative and nondirect costs and thus avoid gross underestimates of total cost. All costing parameters were summed to determine the overall annual cost associated with operating the KBTH pediatric oncology treatment center.

### Cost-effectiveness Analysis

Cost-effectiveness was calculated using the cost per new diagnosis combined with the estimated 5-year survival, thus allowing the estimation of the cost per life saved (Table 1 summarizes the key parameters). Currently, KBTH is only able to track survival for 1 year from diagnosis; at this time period, 58.6% of patients were still alive. To estimate the proportion of patients alive at 5 years from diagnosis, comparable literature was used. For example, a study in Chennai, India found that 5-year survival in a lower- to middle-income setting was 62% of 1-year survival overall for childhood cancers.^13^ We used this same proportion to convert the 1-year survival at KBTH to a 5-year survival of 35%. Given the uncertainty in this estimate, we conducted sensitivity analyses reducing the 5-year survival to 30%.

**Table 1:**
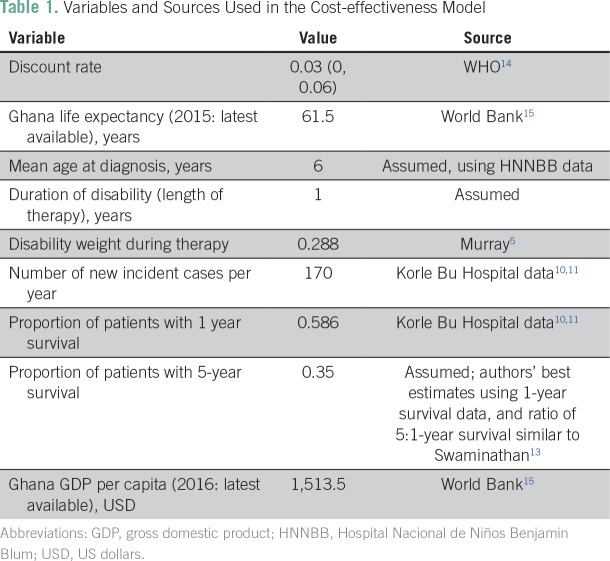
Variables and Sources Used in the Cost-effectiveness Model

The cost per life saved was then converted to cost per disability-adjusted life-years (DALYs) averted using Ghana’s life expectancy of 61.5 years,^15^ with the mean age at diagnosis of 6 years. Although length of treatment of individual childhood cancers varies, we used 1 year as the median duration of therapy, because lymphomas, retinoblastoma, and Wilms tumors constituted a major portion of cancers treated locally. During treatment, children suffer from diminished quality of life; we accounted for this using the Global Burden of Disease disability weight of 0.288.^6^

As recommended using the WHO–Choosing Interventions That Are Cost-Effective (CHOICE) guidelines, discounting was incorporated with a base case of 3%.^16^ Additional estimates using 0% and 6% were also calculated. To estimate the effects of long-term chronic conditions and premature mortality, we completed a one-way sensitivity analysis. The number of additional years survived after diagnosis was varied by allowing a 15% and a 30% reduction of additional years of life expected at age 6 years (ie, base case was survival to normal life expectancy for Ghana of 61.5 years, with variants being survival to age 53.5 and 45.5). The proportionate reduction of life expectancy for cancer survivors was based on data for the United States,^17,18^ given the lack of comparable data for sub-Saharan Africa. It is important to note that this is likely a conservative assumption, given that treatment intensity is far lower in a Ghanaian setting, and most reduction in life expectancy for survivors in HICs is due to treatment-related effects, such as cardiopulmonary dysfunction and second malignant neoplasms.^17^

We used the WHO thresholds for cost-effectiveness.^19^ These thresholds suggest that interventions costing less than per capita income per DALY averted are very cost effective and those costing less than three times per capita income per DALY averted are cost effective. In 2016, the World Bank^15^ listed Ghana’s per capita gross national product as USD $1,513.

## RESULTS

**Fig 1:**
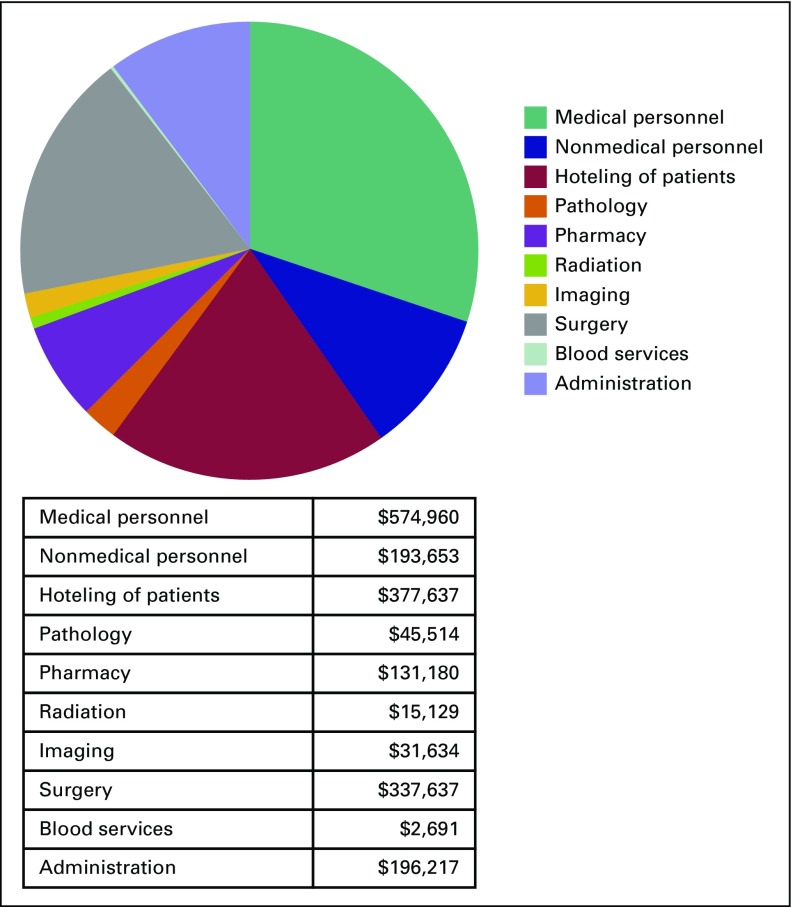
Annual costs of operating a pediatric oncology service.

The annual cost to operate a pediatric cancer unit in Accra, Ghana was estimated as $1.7 million for the 2016 to 2017 year. On the basis of admissions data at KBTH, this equates to $9,781 per pediatric patient newly diagnosed with cancer (Table 2; Appendix Table A1). The largest single cost component was personnel (46.2% of costs), followed by the cost of the operating theaters (22.7%; Fig 1). Chemotherapy and supportive medication accounted for 7.9%; hoteling of patients (room and board) for 5.8%; and central administration costs, including utilities, were estimated as 11.8%. Diagnosis-related costs (pathology and laboratory costs as well as imaging) amounted to 4.6%. The balance of costs was attributed to radiation (0.9%) and blood services (0.2%).

**Table 2:**
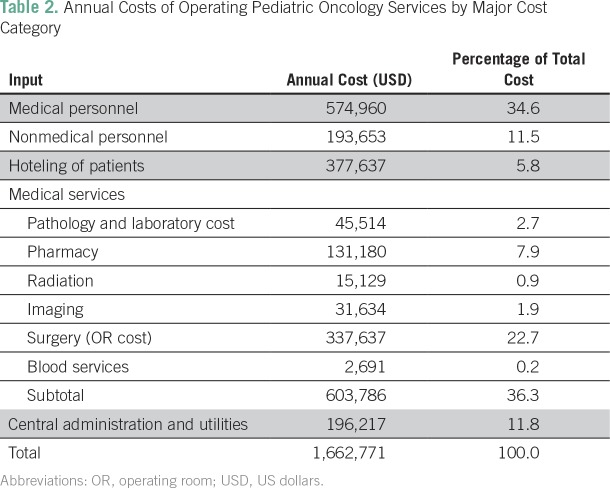
Annual Costs of Operating Pediatric Oncology Services by Major Cost Category

Using the parameters outlined in the base case (Table 1), the cost per life saved was $27,946. The cost per DALY averted in the base case was $1,034, less that Ghana’s per capita income ($1,513), thus meeting WHO-CHOICE criteria for being considered very cost-effective. These results were sustained after adjusting for late effects and early mortality risk at the 0% and 3% discounting levels (Table 3). When 6% discounting was tested, operating the cancer unit remained cost effective.

**Table 3:**
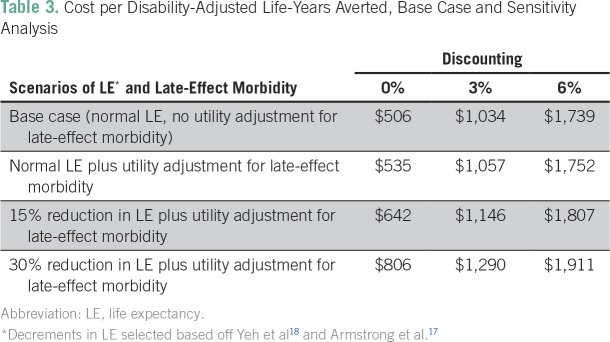
Cost per Disability-Adjusted Life-Years Averted, Base Case and Sensitivity Analysis

Because of uncertainty regarding the 5-year survival of patients with pediatric cancer treated at KBTH, a sensitivity analysis was conducted using 30% reduction in life expectancy and utilities-adjusted late-effect morbidity with 3% discounting. These model parameters represent a conservative upper bound for cost-effectiveness. Under these conditions, when 5-year survival was adjusted down from 35% to 30%, operation of the pediatric cancer unit remained very cost-effective ($1,505 per DALY averted).

## DISCUSSION

The total cost of the pediatric oncology cancer center at KBTH was $1.7 million, or $9,781 per newly diagnosed case per year. The cost per DALY averted was $1,034, meeting the WHO-CHOICE criterion of very cost-effective.

Relatively high shares of personnel costs and relatively low allocations to traded consumables have been previously shown in other aspects of health care in sub-Saharan Africa. A comparative study of pathology and laboratory medicine^20^ found that a major public teaching hospital in Nigeria used < 12% of the annual diagnostic tests per bed as compared with five other public and private hospitals across a range of countries (Kenya, India, Malaysia, and the United States). Presumptive diagnoses without confirmatory tests allow for cost savings on pathology specimens and training. However, the number and cost of consequent erroneous treatments are unknown. Indeed, administering inappropriate treatment regimens is likely to incur significant cost with minimal chance of efficacy. Studies of the cost-effectiveness of improving diagnostic capabilities, both for childhood cancer and other conditions, are warranted.

Similarly, given the high usage of surgery, expenditures on blood services were lower than expected. Parents must pay out of pocket for many medical services, except when support is available from private foundations, with consequent service underutilization relative to countries with more comprehensive health insurance coverage. The use of less-intense chemotherapy protocols to avoid toxicities and reduce supportive care requirements in LMICs such as Ghana is another major reason for relatively low chemotherapy costs.

Other warranted interventions may include those targeting abandonment of treatment, which contributes to a substantial portion of treatment failures in childhood cancer in LMICs.^2,21,22^ Abandonment of treatment can occur for many reasons, including financial hardship and a lack of awareness of the disease and necessary treatment. At KBTH, charitable organizations like Ghana Parents’ Association for Childhood Cancer and World Child Cancer aim to decrease abandonment rates by raising financial contributions to fund medical services when necessary. In other settings, interventions such as social workers and psychologists, who work alongside families providing emotional and psychosocial support, have dramatically decreased abandonment rates.^21^ Although such interventions would increase the center’s operating costs, resultant improvements in treatment adherence may translate to increased cost-effectiveness. The cost-effectiveness of specific abandonment interventions awaits further study.

Despite the above factors, our main finding was that the delivery of pediatric cancer services was very cost effective using WHO-CHOICE definitions. Even in sensitivity analyses using more pessimistic assumptions of 5-year survival and life expectancy, pediatric cancer treatment remained very cost effective. However, cost-effectiveness is distinct from affordability. The annual cost of treating a patient newly diagnosed with cancer is more than six times the Ghanaian per capita gross domestic product, even with modest expenditures on diagnostics, chemotherapy, and radiation.

Historically, health system priorities in many LMICs have focused on the treatment of communicable diseases along with maternal and infant mortality. This has been justifiable, given the historically high burden of such diseases and highly cost-effective interventions, such as vaccines and HIV prevention strategies.^23,24^ However, as strides against communicable diseases are made and countries undergo demographic transitions, disease burdens shift from communicable to noncommunicable ones.^3^^,^ Many LMIC health systems grapple with the changing health care needs that accompany these epidemiologic shifts. Even in countries with universal health insurance, the degree of coverage of noncommunicable diseases often varies. In Ghana, the National Health Insurance Authority does not currently cover childhood cancer treatment, leaving families with the burden of financing and prone to catastrophic health expenditures. By contrast, progress has been made in Ghana in the public financing of specific adult cancers, with coverage of breast and cervical cancer treatment. Interestingly, a Ghanaian study in 2012 found that biennial screening clinical breast examinations coupled with treatment were associated with a cost per DALY averted of $1,299,^25^ a figure slightly higher than that associated with childhood cancer treatment in this study. This knowledge can inform context-sensitive decision making about resource allocation toward childhood cancer among competing health priorities on the part of Ghanaian policymakers, as was recently done in Mexico.^26^ Of note, childhood cancer programs in LMICs have demonstrated the ability to attract funding from alternative philanthropic sources,^8^ allowing for more children to potentially access treatment without detracting from other areas of need.

Several limitations merit note. First, we were unable to determine the specific costs for nonmedical personnel and for central administration and were instead forced to rely on estimates from El Salvador. Second, many costs were determined by extrapolating focal periods of data collection to annual figures. This may not account for fluctuations in volume or intensity over the course of a year, adding an additional degree of uncertainty to our final estimates. Our approach, however, balances true microcosting with feasibility in settings with limited data resources.

Finally, we did not include indirect costs borne by families. Financial toxicity resulting from out-of-pocket costs is significant among HIC caregivers of children with cancer and LMIC adult patients with cancer.^27,28^ These costs are not well characterized in LMIC pediatric oncology but are likely significant.^29^ Incorporating these would thus raise the overall cost of treating childhood cancer. Nonetheless, all the above limitations are unlikely to change our finding of the KBTH childhood cancer unit being very cost-effective, a finding unchanged in even our most conservative sensitivity analysis. Indeed, despite these limitations, this study nonetheless provides the most rigorous data to date for a childhood cancer unit in sub-Saharan Africa.

We outline the total cost of maintaining a childhood cancer treatment center in Ghana and demonstrate that treating childhood cancer is very cost-effective. Similar studies in other LMIC centers of increasing complexity are warranted, as are cost-effectiveness analyses of specific interventions within such centers. The results of this study can be used to inform policy decisions to strengthen child cancer outcomes in sub-Saharan African and other LMICs.

## Figures and Tables

**Table A1:**
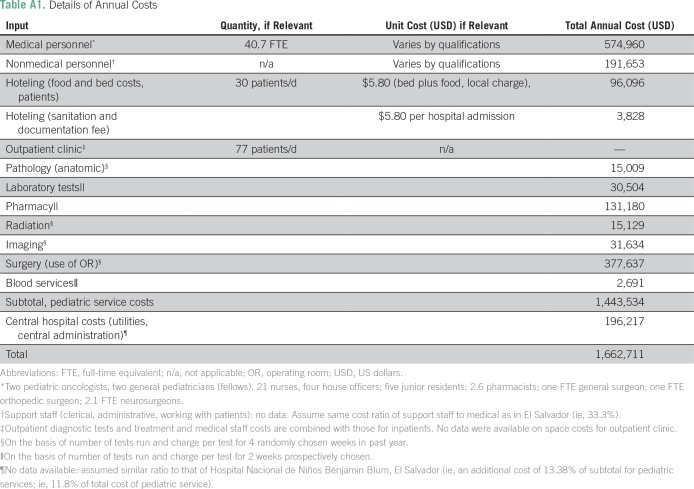
Details of Annual Costs
